# The Role of FeNO in Predicting Asthma

**DOI:** 10.3389/fped.2019.00041

**Published:** 2019-02-21

**Authors:** Mariëlle W. Pijnenburg

**Affiliations:** Division of Respiratory Medicine and Allergology, Department of Pediatrics, Erasmus MC-Sophia, University Medical Center Rotterdam, Rotterdam, Netherlands

**Keywords:** exhaled nitric oxide, preschool children, asthma, diagnosis, prediction, lung growth

## Abstract

Asthma-like symptoms like wheezing and dyspnea affect 1 in every 3 preschool children. An easily available biomarker that predicts later asthma or unfavorable lung growth in these children may be helpful in targeting the right child with the right drugs and avoiding exposure to potentially harmful drugs in others. The fraction of exhaled nitric oxide (FeNO) has been suggested as a marker of eosinophilic inflammation. FeNO can be measured in a standardized way from the age of 4 but several methods have been developed to measure FeNO also in younger children. Several studies have assessed the predictive value of FeNO in preschool wheezing children for asthma later in life. These studies have shown that FeNO may be helpful in defining different preschool wheezing phenotypes, and in assessing the risk of later asthma or impaired lung growth. However, data are conflicting on the added value over clinical parameters. In two studies in school children, high FeNO was predictive for asthma development during follow up and also predicted lower lung function growth. In school children with respiratory symptoms suggestive of asthma, particularly in atopic children, FeNO has diagnostic value for an asthma diagnosis, mostly for ruling in asthma. There are not enough data to assess if FeNO has a predictive value for lung development in school children.

## Introduction

Asthma is the most prevalent chronic disorder in the western world affecting 5–15% of all school aged children and most of them have their first respiratory symptoms before they turn 5 (1, 2). Asthma-like symptoms like wheezing and dyspnea are even more frequent in preschool children with 1 in every 3 children having at least 1 wheezing episode before the age of 4 (3). Only a minority of these preschool children will develop asthma later in life and may benefit from early treatment with controller therapy to prevent symptoms and exacerbations (4). However, some of them, in particular children with severe wheezing exacerbations during the preschool years, have a lower lung function already at age 5 or 6 (5, 6). As lung function tracks during life, it is important to identify children at risk for developing asthma, impaired lung growth, and/or early decline in lung function as early as possible (7). Although the natural course of asthma cannot be altered by asthma medications such as the inhaled corticosteroids (ICS), symptoms can be reduced and lung function possibly maintained while on ICS (8, 9). Therefore, an easily available non-invasive biomarker that predicts later asthma in preschool children may be helpful in targeting the right child with the right drugs and avoiding exposure to potentially harmful drugs in others.

The fraction of exhaled nitric oxide (FeNO), has been suggested as a non-invasive biomarker of (mostly eosinophilic) inflammation (10). In atopic asthmatic children, there is a moderate to goodcorrelation between FeNO and eosinophil counts in blood, induced sputum or broncho-alveolar lavage fluid (10–12). In preschool children elevated blood eosinophils have been associated with the development of later asthma suggesting that blood esoinophils may be an easy biomarker for later asthma (13, 14).

FeNO can be measured in a standardized way with an online single breath technique with constant expiratory flow, with increasing success rates of 40% at age 4 to almost 100% at age 10 (15, 16). Unfortunately, in preschool children the single breath technique is not feasible and alternative techniques have been developed for FeNO measurement (15). The tidal breathing offline method, with collection of exhaled air via a facemask in an appropriate reservoir for later analysis, is the most simple, is feasible in almost all children and shows good within-subject reproducibility (17, 18). The disadvantages of this technique are that expiratory flow is not controlled, while FeNO is highly flow-dependent, and that contamination with high concentrations of NO produced in the nasal cavity cannot be avoided. The latter can be solved by exhaling against a resistance and with a septum in the facemask, separating air from the upper and lower airways. A modification of the online single breath technique is to measure FeNO during a (raised volume) rapid thoracic compression maneuver at the end-expiratory plateau phase of the NO profile (19). This technique requires sedation and highly trained personnel. Alternatively, in children who do accept a mouthpiece manual adjustment of the expiratory flow or the use of dynamic flow restrictors may allow for a single breath, flow-controlled exhalation (20, 21).

Newer technology with fast-response chemiluminescence analyzers and flow control devices, may make it feasible to measure FeNO with a single breath technique in children as young as 3 years (22). Also, with mathematical algorithms single breath constant flow values may be derived from tidal breathing FeNO values (23). For now, the offline tidal breathing technique with a mask separating mouth and nose seems the most simple method to measure FeNO in preschool children.

### Predicting Asthma With FeNO

Several studies assessed FeNO in preschool children. Higher FeNO values were found in young children with (recurrent) wheezing compared to healthy preschool children (19, 24–26), children with frequent wheezing with high vs. low asthma predictive index (API) (27, 28) and in children with persistent wheezing vs. transient wheezing (29). These studies were mainly cross sectional and compared two groups of children. Although these studies suggest that FeNO may help to phenotype young wheezing children, only few studies assessed the predictive value of FeNO in the preschool years for later asthma.

In a study in almost 400 children with lower airway symptoms, Singer et al. measured FeNO offline with a tidal breathing technique at a mean age of 22 months, and modified the classical API by including FeNO > 10 ppb as a major criterion and changing blood eosinophils > 4% from major to minor criterion (30). At school age, a doctor's diagnosis of asthma was based on standardized interviews. Children with asthma at school age had higher FeNO levels compared to children without asthma at that age (10.5 vs. 6.2 ppb; *P* < 0.001). The new API including FeNO had a positive predictive value of 58% and a negative predictive value of 78% for later asthma, which was actually comparable to the classical API.

Caudri et al. studied the predictive value of FeNO, specific IgE and airway resistance as measured with the interrupter technique (Rint) in 3–4 year old children with wheezing symptoms (31). Both FeNO and specific IgE were associated with asthma at 8 years, also after mutual adjustment and adjustment for clinical history. In other words, FeNO had an additive predictive value over specific IgE and also over the clinical history (asthmatic mother, wheezing frequency and eczema). This study showed that even though FeNO and specific IgE are correlated, FeNO does not merely reflect atopy.

A cross sectional study from Denmark showed low feasibility of online single-breath FeNO measurements ranging from 0 to 71% in children of 3 to 6 years old, respectively (32). In this study FeNO >11.5 ppb had low sensitivity but high specificity (93%) for an asthma diagnosis.

In unselected newborns FeNO before any respiratory symptom occurred was not associated with asthma at school age, suggesting that environmental factors are needed to induce NO production (33). However, in a longitudinal study in 116 high-risk children with eczema, Chang and co-workers found increased FeNO levels before any wheezing illness in children who had asthma and increased airway reactivity at age 5 (34). A steeper increase in FeNO was seen in children with asthma at age 5 compared to children without asthma at that age, which is in line with the hypothesis that environmental factors are needed to induce iNOS and that there might be a window of opportunity in the preschool years.

In a longitudinal cohort of 42 preschool children (6–24 months old) with recurrent wheezing FeNO ≥ 30 ppb as assessed with a single breath technique during the raised volume rapid thoracic compression technique had a high predictive value for persisting wheezing at age 3 (area under the receiver operating curve, AUC 0.86) (35). This predictive value was better than that of the API. What is more, FeNO ≥ 30 ppb but also tidal-FENO ≥ 7 ppb was associated with a decline in FEV_0.5_ and FEF_25−75_ between inclusion and age 3 years. While using the same single breath technique, the same group, showed that a 10 ppb increase in FeNO was associated with a 0.4 z-score decline in FEV_0.5_, a 0.4 z-score decline in FEF_25−75_, and a 0.42 z-score decline in FEF_75_ 6 months later (36). FeNO also predicted wheezing exacerbations in the 6 months following measurement (AUC 0.83, 95% CI: 0.69–0.96). However, measuring single breath FeNO in infants and preschool children is challenging, requires well-trained personnel and is only feasible in research settings.

Klaassen et al. assessed if a change in FeNO after 8 weeks of inhaled corticosteroids in preschool children predicted asthma at school age but this was not the case (37).

It is important to realize that cut off values and predictive values of FeNO may differ between the studies due to different measurement techniques.

In school children only 2 studies have been published examining the predictive value of FeNO for the development of asthma. A study in more than 2,000 asthma-free school children (aged 7–10 years) assessed if FeNO could predict asthma development during 3 years follow up. The authors showed that children in the highest FeNO quartile had more than a 2-fold increased risk of new-onset asthma compared to those with the lowest quartile (hazard ratio 2.1, 95% CI 1.3–3.5). This effect was most notable in children with a negative parental history of asthma and did not vary with a history of allergic rhinitis (38). In a smaller study 109 children with allergic rhinitis (mean age 8.4 years, range 7–13) were followed for 5 years when they were evaluated for an asthma diagnosis (39). Children with FeNO > 35 ppb at baseline had a higher risk of developing asthma when compared to children with lower FeNO values (OR 2.49, *P* < 0.01) and every 10 ppb increase above this cutoff doubled the risk of asthma further. Also, children with FeNO > 35 ppb had a decrease in FEV_1_ and FVC while in the children with FeNO < 35 ppb FEV_1_ and FVC increased, suggesting less lung growth in children with high FeNO values.

In summary, the studies mentioned here have shown that FeNO may be helpful in objectively defining different wheezing phenotypes in preschool children, and assessing the risk of asthma or impaired lung growth later in life. Preschool children with increased FeNO levels have an increased risk of later asthma. Data are conflicting on the added value over clinical parameters. In two studies in school children, high FeNO was predictive for asthma development during follow up and also predicted lower lung function growth.

### FeNO in the Diagnosis of Asthma in School Children

In children only few studies assessed the value of FeNO for diagnosing asthma (40–42). Woo et al. included 245 steroid-naïve children with respiratory symptoms suggestive of asthma. Asthma was diagnosed in 167 of them, based on symptoms and ≥ 12% bronchodilator response and/or airway hyperresponsiveness as assessed with methacholine provocation (40). The negative and positive predictive values of FeNO at a cutoff of 22 ppb for an asthma diagnosis were 90.5 and 48.6%, respectively. FeNO performed better as a diagnostic test in the atopic children (AUC 0.85, 95% CI 0.79–0.90) if compared to atopic and non-atopic children all together (AUC 0.76; 95% CI 0.70–0.82). In a study from Israel in 150 children who were referred with respiratory symptoms, FeNO, sputum eosinophils and FEV_1_ were assessed at baseline. During the following 18 months asthma was diagnosed based on wheezing exacerbations, bronchodilator response ≥ 15% and/or FEV_1_ variability ≥ 15% and/or a positive bronchoprovocation test (41). FeNO performed similar as sputum eosinophils for the diagnosis asthma (AUC 0.90 and 0.92, respectively) and better than baseline FEV_1_. Nineteen ppb appeared the best cutoff with sensitivity of 80%, specificity of 92%, positive predictive value of 89% and a negative predictive value of 86%.

A third study that assessed the diagnostic value of FeNO was performed in 4–8 years old children with asthmatic symptoms. Children with recent recurrent wheeze were labeled as “probable asthma” and compared to controls. The analysis of the ROC showed that FeNO had good power for discriminating between children with probable asthma and healthy controls, with a sensitivity of 86% and specificity of 92% at the cut off level of 1.5 SD above predicted. This was better than the predictive value of lung function and bronchodilator response as assessed with impulse oscillometry (42).

A real-life retrospective study in 3,612 children with symptoms suggestive of asthma showed poor predictive value of FeNO >15.8 ppb for an asthma diagnosis with an area under the ROC curve of only 0.53 (43).

In an unselected population of 1,602 children, questionnaires, spirometry with bronchodilator response and FeNO were assessed. Asthma was defined as symptoms suggestive of asthma with reported medical diagnosis or evidence of bronchodilator response. FeNO had poor predictive value (area under the ROC curve 0.62) (44). This poor predictive value may be explained by the fact that unselected children were included, in contrast to most studies that included children with respiratory symptoms.

In general, FeNO has diagnostic value for an asthma diagnosis, in particular in children with respiratory symptoms and in atopic children and mostly ruling in asthma. The National Institute for Health and Care Excellence (NICE) recommends measuring FeNO as a diagnostic tool in adults and children who have an intermediate probability of having asthma and in combination with other diagnostic options such as spirometry (45). Similarly, the British Thoracic Society guidelines recommend to use FeNO (if available) “to find evidence of eosinophilic inflammation” (46). In contrast, the 2018 GINA guidelines conclude that FENO has not been established useful for an asthma diagnosis (4).

### FeNO in Predicting Lung Function Decline

Several recent studies showed that high baseline FeNO was associated with reduced lung function growth in asthmatic children (47) and accelerated lung function decline in adults (48, 49). In the pediatric study from China 193 asthmatic children, mean age 9.7 years (SD 1.9 years) were followed for 5 years (47). One quarter had reduced lung function growth which was associated with lower baseline spirometry, higher FeNO and female sex. Also, two single nucleotide polymorphisms (GSDMB_rs2305480 and IL33_rs1342326) were associated with longitudinal changes in lung function.

More longitudinal studies in children are needed to assess the predictive value of FeNO for lung development.

## Conclusion

In preschool children FeNO may be helpful in objectively discriminating between different wheezing phenotypes and in predicting asthma later in life.

Only few studies in school children assessed the diagnostic value of FeNO for an asthma diagnosis; particularly in atopic children with respiratory symptoms, FeNO has a high positive predictive value for an asthma diagnosis. If FeNO helps to predict lung function decline or impaired lung growth remains to be shown.

## Author Contributions

The author confirms being the sole contributor of this work and has approved it for publication.

### Conflict of Interest Statement

The author declares that the research was conducted in the absence of any commercial or financial relationships that could be construed as a potential conflict of interest.
